# Genome-Wide Identification of the Plasma Membrane H^+^-ATPase in Poplar and Their Responses to Abiotic Stresses and Nitrogen Availability

**DOI:** 10.3390/life16101691

**Published:** 2026-10-09

**Authors:** Jiayi Gu, Limengjie Wang, Bingjing Li, Lianke Guan, Hang Wang, Kebing Du, Jie Luo

**Affiliations:** 1 Hubei Engineering Technology Research Center for Forestry Information, College of Horticulture and Forestry Sciences, Huazhong Agricultural University, Wuhan 430070, China; gu1234112025@163.com (J.G.); limengjie_wang@163.com (L.W.); 18222457031@163.com (B.L.); lk_guan@163.com (L.G.); kebingdu@mail.hzau.edu.cn (K.D.); 2 Key Laboratory of Cultivation and Utilization for Oil-Camellia Resources, Experimental Center for Subtropical Forestry, Chinese Academy of Forestry, Xinyu 336600, China; wangghangg@163.com

**Keywords:** abiotic stress, H^+^-ATPase, nitrogen, *Populus*, transcriptome analysis

## Abstract

Plasma membrane (PM) H^+^-ATPase (HA) plays critical roles in plant growth, development, and stress responses, whereas few studies have focused on the *HA* gene family in poplar. In this study, 13 *HA* genes were identified from each of the two haplotype genomes of 717 poplar (*Populus tremula* × *P. alba* 717-1B4), and all identified HA proteins in 717 poplar contained three conserved functional domains. *Cis*-element analysis revealed that promoters of 717 poplar *HA* genes contained a large number of light-, stress-, and hormone-responsive *cis*-elements. Transcriptome data showed that *P. trichocarpa PtrHA5* and *PtrHA7* specifically responded to short-term heat and long-term drought stress in stems and roots, respectively, while *PtrHA3* and *PtrHA10* responded to multiple abiotic stresses in roots, stems, and leaves. In 717 poplar, *PtaHA9* was significantly down-regulated in roots under low-nitrate conditions and up-regulated under high-nitrate conditions. Co-expression network analysis in *P. trichocarpa* indicated that *PtrHA9* had the largest number of co-expressed genes and shared overlapping co-expressed genes with other *PtrHA* genes. GO and KEGG enrichment analyses of genes within the co-expression network suggested that genes co-expressed with *PtrHAs* were enriched in energy metabolism, enzyme-activity regulation, and transmembrane substance transport. Collectively, this study systematically characterized the evolutionary and expression profiles of the *HA* gene family in 717 poplar and suggests putative functional associations of *HA* genes with poplar stress adaptation.

## 1. Introduction

The *H^+^-ATPase* (*HA*) gene family, widely present in diverse terrestrial plants, plays indispensable and critical roles in regulating plant growth, development, and abiotic-stress adaptation [1]. This gene family, which belongs to the P-type ATPase family, specifically encodes plant plasma membrane (PM) HA with highly conserved gene sequences and protein structures across species, laying an important foundation for functional stability [2]. In terms of protein structural features, HA proteins fold from a single polypeptide chain of approximately 100 kDa, typically containing ten transmembrane domains (TDs) [2]. The core catalytic domain resides between TD4 and TD5, whereas both the N- and C-termini of the polypeptide are exposed to the cytosol; such spatial structural features underpin its proton-transport function [3,4]. Physiologically, HA couples ATP hydrolysis to proton transmembrane transport to generate a transmembrane proton-motive force that activates secondary transport systems, thereby coordinately modulating multiple plant physiological processes, including stomata opening, nutrient uptake, cytosolic pH homeostasis, as well as adaptation to abiotic stresses [5]. As a key regulator of cellular pH homeostasis, HA exhibits an optimum pH below 7.0, which mediates H^+^ efflux to maintain cytoplasmic pH within a normal physiological range [6]. Relying on the stable proton electrochemical gradient, HA drives the transmembrane uptake and transport of ions, sugars, amino acids, and other nutrients to sustain intracellular ion and metabolic balance [1,7,8]. Blue light activates PM HA in guard cells, triggering charge accumulation and increased guard-cell turgor to induce stomatal opening [9]. Furthermore, PM H^+^-ATPase activation globally up-regulates transcript levels of cell wall modification-related genes, thereby promoting cell growth and shoot growth in light-grown Arabidopsis seedlings [10].

In addition to regulating normal plant growth and development, *HA* also mediates plant resistance and adaptation to multiple abiotic stresses, including extreme temperature, drought, and high salinity [1,5]. Short-term cold treatment suppresses *Arabidopsis* PM ATPase activities, whereas prolonged cold leads to substantial reactivation of their activities [11]. In cucumber, the abscisic acid (ABA)/H_2_O_2_ signaling cascade triggers transcriptional up-regulation of *CsHA4* and *CsHA8* in response to heat stress [12]. Under drought stress, reduced protein abundance of CALCIUM-DEPENDENT PROTEIN KINASE RELATED KINASE 1 (ZmCRK1) alleviates phosphorylation-mediated repression of *ZmMHA2* at Ser^901^, thereby promoting *ZmMHA2*-dependent H^+^ efflux to drive stomatal closure and enhance drought adaptation [13]. HA activities are dynamically modulated according to drought intensity in leaves of *Populus euphratica*; in moderate drought, HA activities remain nearly unchanged; however, under severe drought, HA activities decrease to reduce ATP consumption, which, together with stomatal closure and selective ion accumulation, contributes to drought tolerance [14]. In poplar, several genes can improve salt or drought tolerance, such as *MYB DOMAIN PROTEIN 73A* (*PagMYB73A*), *HOMEOBOX 7a* (*PagHB7a*), and *XYLOGLUCAN ENDOTRANSGLUCOSYLASE/HYDROLASE 12* (*PagXTH12*) [15,16,17]. Furthermore, HA establishes a transmembrane proton-motive force to drive the PM Na^+^/H^+^ antiporter SALT OVERLY SENSITIVE 1 (SOS1) for cytosolic Na^+^ extrusion, alleviating Na^+^-triggered ionic toxicity under salt stress [5,18]. Salt stress significantly up-regulates *AHA1* and *AHA2* in *Arabidopsis thaliana* [19]. Heterologous overexpression of sunflower (*Helianthus annuus*) *HHA4* and *HHA11* also enhances plant salt tolerance [20]. In *P. euphratica*, *HA1* is activated by WRKY DNA-BINDING PROTEIN 1 (WRKY1) under salt stress, and elevated PM proton-pump activities confer improved salt resistance [21].

Beyond its roles in plant stress defense, the proton-transport function of the *HA* gene family is also critical for plant adaptation to nutritional stress [22]. Under low-nitrogen conditions, *Brassica napus* showed increased nitrogen use efficiency by enhancing nitrogen uptake via remodeling root system architecture and regulating PM H^+^-ATPase activities [23]. In spruce, root HA proteins continuously extrude H^+^ to generate a transmembrane proton-motive force that fuels NH_4_^+^ and NO_3_^−^ symport, constituting an indispensable energy source for efficient inorganic nitrogen uptake [24]. In *P. popularis*, low-nitrogen treatment markedly down-regulates the key *HA* gene *VHA2;2*, accompanied by reduced total HA activities, which may restrict root NH_4_^+^ and NO_3_^−^ influx [25]. Furthermore, inactivation of *HA* activities markedly reduces NH_4_^+^ and NO_3_^−^ uptake by roots of *P. popularis* [25]. Under low-nitrogen stress, *HA* genes homologous to Arabidopsis *AHA2* and *AHA10* are up-regulated in the SI region of the primary root absorption zone in 717 poplar (*P. tremula* × *P. alba* 717-1B4), where they coordinate with ammonium transporters to potentially sustain root NH_4_^+^ acquisition under nitrogen-limited conditions [26].

Poplar species are fast-growing woody plants with substantial economic and ecological value [27,28]. Against the background of global climate change and aggravated soil salinization, poplars frequently suffer from multiple abiotic stresses such as salinity and drought, which severely constrain their growth and development, as well as afforestation application [29,30]. The *HA* gene family plays central roles in modulating intracellular pH homeostasis, ion balance, substance transport, and stress responses in plants [1] and represents key functional genes enabling woody plants to tolerate abiotic stresses including salinity and drought [31,32,33]. Several *HA* genes have been functionally characterized in other poplar species, such as *PtoPHA14D* in *P. tomentosa* and *PeHA1* in *P. euphratica* [21,34], while none have been characterized in 717 poplar to date. The 717 poplar features high-quality genome assembly and amenability to genetic transformation, making it an ideal model for dissecting gene functions and molecular mechanisms underlying stress tolerance in poplars [28,35,36]. Accordingly, this study performed genome-wide identification of the *HA* gene family in 717 poplar and characterized their gene structures, conserved domains, gene duplication events, and *cis*-regulatory elements. Transcriptome data were integrated to investigate expression profiles of *HA* genes in poplar species across diverse tissues, developmental stages, and diverse abiotic-stress conditions. Co-expression networks were further constructed, followed by functional enrichment analyses to explore the potential functions and molecular associations of the *HA* gene family in poplar abiotic-stress responses. This work will provide candidate genes and preliminary insights that may support future development of stress-tolerant poplar breeding materials.

## 2. Materials and Methods

### 2.1. Plant Culture and Treatments

In this study, 717 poplar was micro-propagated following an established tissue-culture protocol [37]. Plant culture and treatments have been described in detail in previous publications [38,39]. Briefly, uniformly grown three-week-old rooted micro-propagated plantlets were transplanted into plastic pots filled with fine sand. Plants were cultivated in a growth chamber under a 16 h light/8 h dark photoperiod at a constant temperature of 23 °C with relative humidity maintained at 50–60%. The photosynthetic photon flux density in the growth chamber was maintained at 100 μmol m^−2^ s^−1^. Each pot contained one individual plant and all pots were randomly arranged and repositioned weekly during the treatment period to eliminate positional microenvironmental effects. After one-week acclimatization, plants were randomly divided into five treatment groups and irrigated every three days with 50 mL modified Hoagland’s nutrient (2 mM KH_2_PO_4_, 1 mM MgSO_4_, 25 μM H_3_BO_3_, 2 μM MnSO_4_, 2 μM ZnSO_4_, 0.5 μM CuSO_4_, 0.5 μM Na_2_MoO_4_, 53.7 μM EDTA-FeNa, pH 5.5), to which calcium nitrate was supplemented as the sole nitrogen source to establish five nitrogen treatments: 0.2, 0.5, 2, 5, and 10 mM nitrate. The 2 mM nitrogen treatment was established as the control, following similar previous studies on poplars [40,41,42]. Calcium chloride was used to balance calcium concentrations across all groups to ensure identical calcium concentration. After a 45-day nitrogen treatment, whole-root tissues were harvested between 8:00 and 11:00 a.m. Samples were wrapped in aluminum foil, immediately frozen in liquid nitrogen, fully ground into fine powder under liquid-nitrogen conditions, and stored at −80 °C.

### 2.2. Identification of HA Genes in 717 Poplar

The accession identifiers of *A. thaliana AtHA* genes from the previous publication [43] were retrieved, and the corresponding AtHA protein sequences were downloaded from The Arabidopsis Information Resource (TAIR, https://www.arabidopsis.org/, accessed on 21 October 2024) [44]. Using these AtHA protein sequences as queries, BLASTP searches were performed via the “Several Sequences to a Big File” tool in TBtools-II (v2.099) [45]. Sequences with an E-value < 10^−5^ were retained. Hidden Markov model (HMM) files of E1–E2 ATPase (PF00122), N-terminus cation transporter/ATPase (PF00690), and haloacid dehalogenase-like hydrolase domains (PF00702) were downloaded from the InterPro database (https://www.ebi.ac.uk/interpro/, accessed on 24 October 2024) [46]. HMMER (version 3.0, http://www.hmmer.org/) [47] was used to search against the 717 poplar protein database [28] with the above HMMs, and sequences with an E-value < 10^−5^ were kept. Candidate *HA* family genes of 717 poplar were obtained by taking the intersection of sequences retrieved from BLASTP and HMMER searches. To distinguish genes derived from the *P. tremula* and *P. alba* haplotypes, gene prefixes *Pt* and *Pa* were assigned for nomenclature, respectively.

### 2.3. Bioinformatic Analyses

The Protein Parameter Calc tool in TBtools-II [45] was used to characterize the basic physicochemical properties of *HA* family proteins in 717 poplar. Chromosomal location information of each *HA* gene was extracted using the GXF Gene Position & Info Extract program combined with genome gff3 annotation files in TBtools-II [45]. Subcellular localization of HA proteins was predicted via the online WoLF PSORT tool [48]. Conserved domains of HA proteins were predicted using the InterPro online database [46]. The TSV output file of domain predictions and the phylogenetic tree file generated by MEGA11 [49] were imported into the Visualize Pfam Result module of TBtools-II [45] for visualizing conserved domains. Meanwhile, the genome gff3 annotation file and phylogenetic tree file were loaded into the Gene Structure View (Advanced) tool of TBtools-II [45] to visualize gene structures.

### 2.4. Phylogenetic Tree Construction

To construct the phylogenetic tree of *HA* genes, HA protein sequences from *A. thaliana*, soybean, rice, maize, and *P. trichocarpa* were retrieved from published studies [50]. Multiple sequence alignment of HA proteins was performed using the ClustalW program in MEGA11 [49]. The phylogenetic tree was constructed using the neighbor-joining method with default parameters, and branch support was estimated with 1000 bootstrap replicates. The phylogenetic tree was further visualized and polished using the iTOL online tool [51].

### 2.5. Cis-Acting Element Analysis and Gene Duplication Event Analysis

The 2000 bp promoter sequences of *HA* genes were extracted using TBtools-II [45]. Potential *cis*-acting elements within these promoters were predicted via the PlantCARE online server (https://bioinformatics.psb.ugent.be/webtools/plantcare/html/, accessed on 29 November 2024) [52].

Gene duplication events of *HA* genes in 717 poplar were analyzed using the One-Step MCScanX tool in TBtools-II [45]. A Circos plot illustrating *HA* gene duplication events was generated with the Advanced Circos tool in TBtools-II [45]. The Multiple Synteny Plot tool of TBtools-II [45] was used to draw synteny plots of *HA* genes between the *P. alba* haplotype of 717 poplar, *A. thaliana*, and rice. Finally, the Simple Ka/Ks Calculator tool in TBtools-II [45] was applied to calculate nonsynonymous/synonymous substitution rate (Ka/Ks) ratios for duplicated gene pairs.

### 2.6. RNA Extraction and RNA-Seq

Root samples of 717 poplar were collected, and total RNA was isolated following the CTAB method described in a previous report [53]. Two samples derived from the same treatment were pooled at equal mass to produce three independent biological replicates. RNA quality was verified by agarose gel electrophoresis and micro-spectrophotometric detection. Qualified total RNA samples were sent to Shanghai Majorbio Bio-Pharm Technology Co., Ltd. (Shanghai, China) for transcriptome library construction and sequencing. Three independent libraries for each treatment were constructed, and RNA-seq was performed on the DNBSEQ-T7 sequencing platform (MGI Tech Co., Ltd., Shenzhen, China) with 150 bp paired-end reads.

### 2.7. Gene Expression Analysis

Expression patterns of poplar *HA* genes, including tissue-specific expression and responses to abiotic stresses and nitrogen availability, were analyzed by integrating publicly available poplar transcriptomic datasets and in-house transcriptome data generated in this study. Expression values of *HA* genes in *P. trichocarpa* (designated as *Ptr*) across different tissues and growing seasons were retrieved from the Phytozome database (https://phytozome-next.jgi.doe.gov/, accessed on 2 December 2024). Transcriptomes of *P. trichocarpa* under multiple stresses were downloaded from the NCBI database with accession PRJEB19784 [54]. Transcriptome data under varying nitrogen levels were obtained from 717 poplar samples generated in the present experiment. Public sequencing reads were downloaded using the iSeq software (v1.9.2) [55]. Raw reads were pre-processed with fastp (v1.0.0) to remove low-quality reads [56]. Clean reads from public datasets and in-house data were separately mapped to the *P. trichocarpa* reference genome (v4.1) and the *P. alba* haplotype genome of 717 poplar using the Subread software (v2.1.1) [57], respectively. Gene-level read counting was calculated using featureCounts [58]. Differentially expressed genes (DEGs) were identified with the DESeq2 package implemented in R [59]. Genes satisfying the thresholds of absolute values of log_2_fold-change (log_2_FC) above 1 and False Discovery Rate (FDR) < 0.05 were regarded as significantly DEGs. Heatmaps illustrating gene expression levels were generated using the pheatmap R package (v1.0.13) [60].

### 2.8. Co-Expression Network Analysis

Co-expression networks centered on *PtrHA* genes were extracted from co-expression data of *P. trichocarpa* available in the Phytozome database (v14, https://data.jgi.doe.gov, accessed on 2 December 2024). Co-expression relationships between *PtrHA* genes and their co-expressed genes were assigned as edges, and these genes served as nodes. The obtained networks were visualized in Cytoscape (v3.10.3) [61]. Gene Ontology (GO) and Kyoto Encyclopedia of Genes and Genomes (KEGG) functional enrichment analyses for all genes within *PtrHAs*-centered co-expression networks were performed following previously reported procedures [62]. Briefly, all protein sequences of *P. trichocarpa* (v4.1) were annotated for GO and KEGG terms via the EggNOG v6.0 database [63]. A species-specific OrgDb annotation package was constructed in R using the AnnotationForge package. Enrichment analysis was performed using the R package clusterProfiler with default parameters [64]. All annotated genes from the *P. trichocarpa* v4.1 genome were used as the background gene set. Terms with FDR < 0.05 were considered significantly enriched. Bubble plots for enrichment results were plotted using the ggplot2 package in R, as described in earlier publications [65,66].

## 3. Results

### 3.1. Identification and Physicochemical Properties of HA Genes in 717 Poplar

A total of 26 HA proteins were identified from the two haplotypes of 717 poplar, with 13 HA proteins detected in each haplotype (Table S1). Amino acid length, molecular weight, isoelectric point, and other physicochemical characteristics of HA proteins were analyzed (Table S1). The lengths of HA proteins ranged from 944 to 970 amino acids (aa), PtHA4 and PaHA4 were the shortest proteins, whereas PtHA3 and PaHA3 were the longest ones (Table S1). The molecular weights of HA proteins varied from 103.78 to 106.55 kDa, and their isoelectric points ranged from 5.54 to 6.99 (Table S1). All HA proteins exhibited positive grand average of hydropathicity (GRAVY) values, indicating that they are hydrophobic proteins (Table S1). Furthermore, subcellular localization prediction revealed that all HA proteins of 717 poplar were localized to the PM (Table S1), consistent with the physicochemical features of membrane-bound proteins. Chromosomal location analysis showed that *HA* genes in 717 poplar were distributed across seven chromosomes, with chromosome 6 harboring the largest number of *HA* genes, with four genes present in each haplotype of this chromosome (Table S1).

### 3.2. Gene Structure and Phylogenetic Analysis of HAs in 717 Poplar

**Figure 1:**
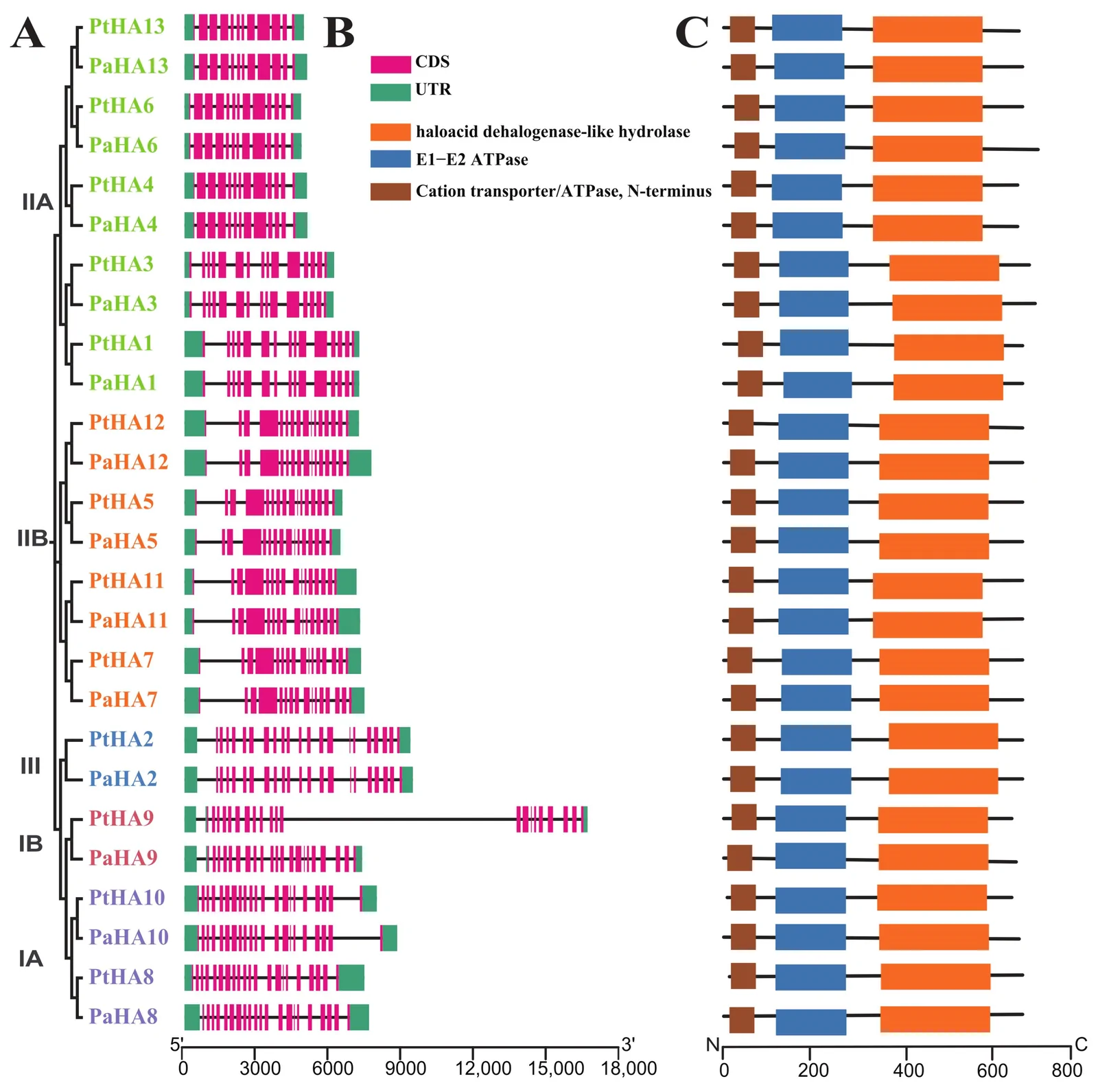
Phylogenetic tree (**A**), gene structure (**B**), and protein domain architecture (**C**) of *HA* genes in 717 poplar. The gene prefixes *Pt* and *Pa* correspond to genes derived from the *P. tremula* and *P. alba* haplotypes of 717 poplar, respectively.

To explore the evolutionary characteristics and structural conservation of the *HA* gene family in 717 poplar, phylogenetic tree construction, gene structure analysis, and conserved domain prediction were performed (Figure 1).

Gene structure analysis revealed that all *PtHA* and *PaHA* genes contained 5′ and 3′ untranslated regions (UTRs) (Figure 1B). In terms of exon distribution, genes in the IIA subfamily contained 12 to 15 exons, and all members of the IIB subfamily had 16 exons (Figure 1B). Consistently, members of the III, IB, and IA subfamilies contained 21 exons (Figure 1B). The gene structures of most *HA* genes contained multiple short introns, exhibiting highly conserved structural patterns within the family (Figure 1B). Notably, *PtHA9* showed distinct structural characteristics from other HA members, containing one long intron combined with multiple short introns (Figure 1B), resulting in a more complex intron composition. Conserved domain analysis demonstrated that all 717 poplar HA proteins harbored three typical conserved domains, including the haloacid dehalogenase-like hydrolase domain, E1–E2 ATPase domain, and N-terminus cation transporter/ATPase domain (Figure 1C), indicating that these three domains may contribute to the biological functions of the 717 poplar *HA* gene family.

**Figure 2:**
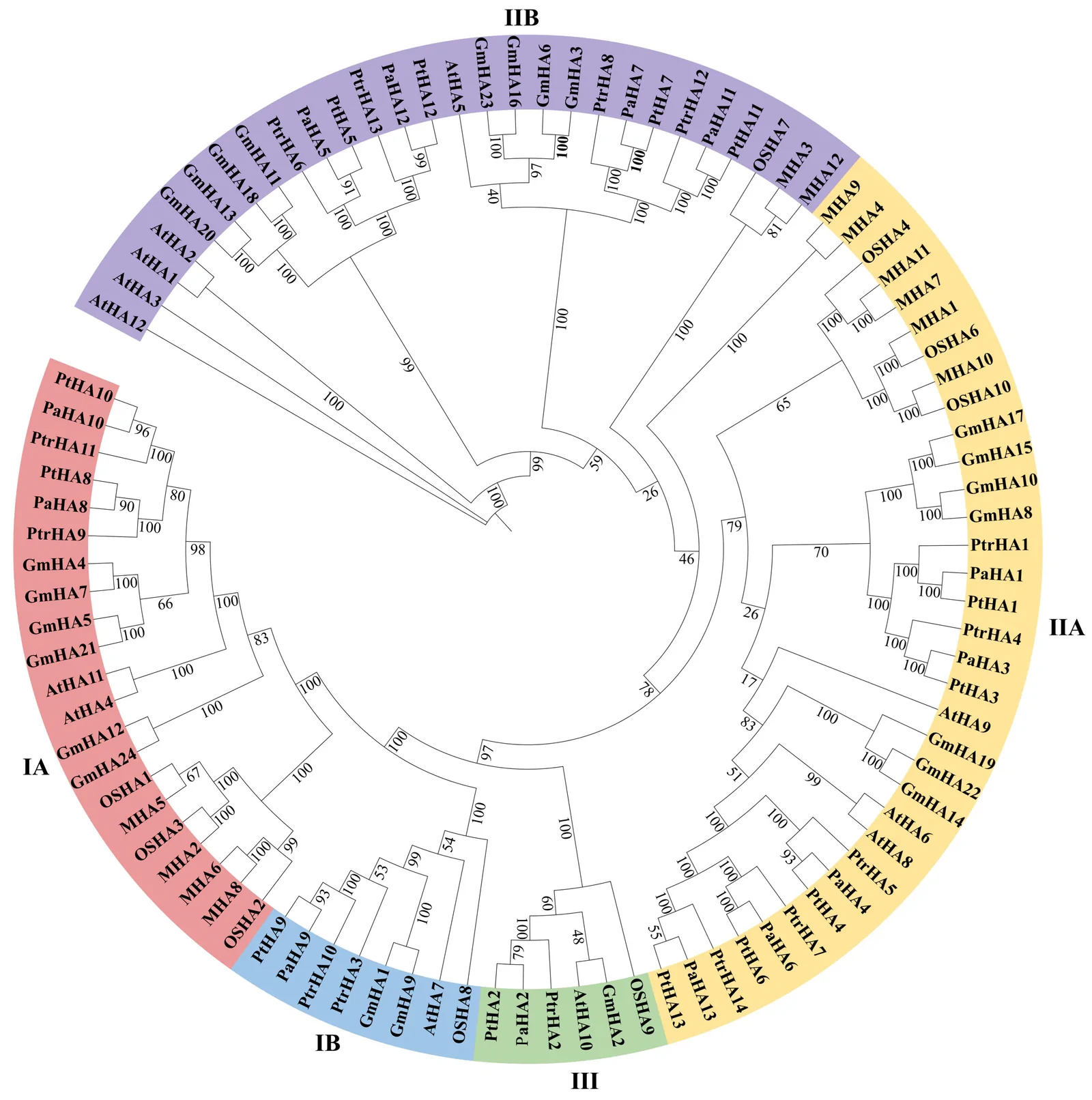
Phylogenetic tree of *HA* genes from multiple species. *At*, *A. thaliana*; *Os*, *Oryza sativa*; *Zm*, *Zea mays*; *Ptr*, *P. trichocarpa*; *Pt*, the *P. tremula*-derived haplotype subgenome of 717 poplar; *Pa*, the *P. alba*-derived haplotype subgenome of 717 poplar; *Gm*, *Glycine max*. Numbers on the branches represent bootstrap support values.

A phylogenetic tree was constructed based on 97 HA protein sequences derived from multiple plant species (Figure 2). According to the phylogenetic relationships, the *HA* gene family was classified into five distinct subfamilies: IA, IB, IIA, IIB, and III (Figure 2). Among them, Pt/PaHA1, Pt/PaHA3, Pt/PaHA4, Pt/PaHA6, and Pt/PaHA13 clustered into the IIA subfamily; Pt/PaHA5, Pt/PaHA7, Pt/PaHA11, and Pt/PaHA12 belonged to the IIB subfamily; Pt/PaHA2 was grouped into the III subfamily; Pt/PaHA9 was classified into the IB subfamily; and Pt/PaHA8 and Pt/PaHA10 belonged to the IA subfamily (Figure 2). However, phylogenetic tree analysis indicated that *PtrHA9* from *P. trichocarpa* was not an ortholog of *PtaHA9* in 717 poplar (Figure 2). Furthermore, the IIA subfamily contained the largest number of HA members (34), whereas the III subfamily contained the fewest members (only six) across all subfamilies (Figure 2).

### 3.3. Analysis of HA Gene Duplication Events

**Figure 3:**
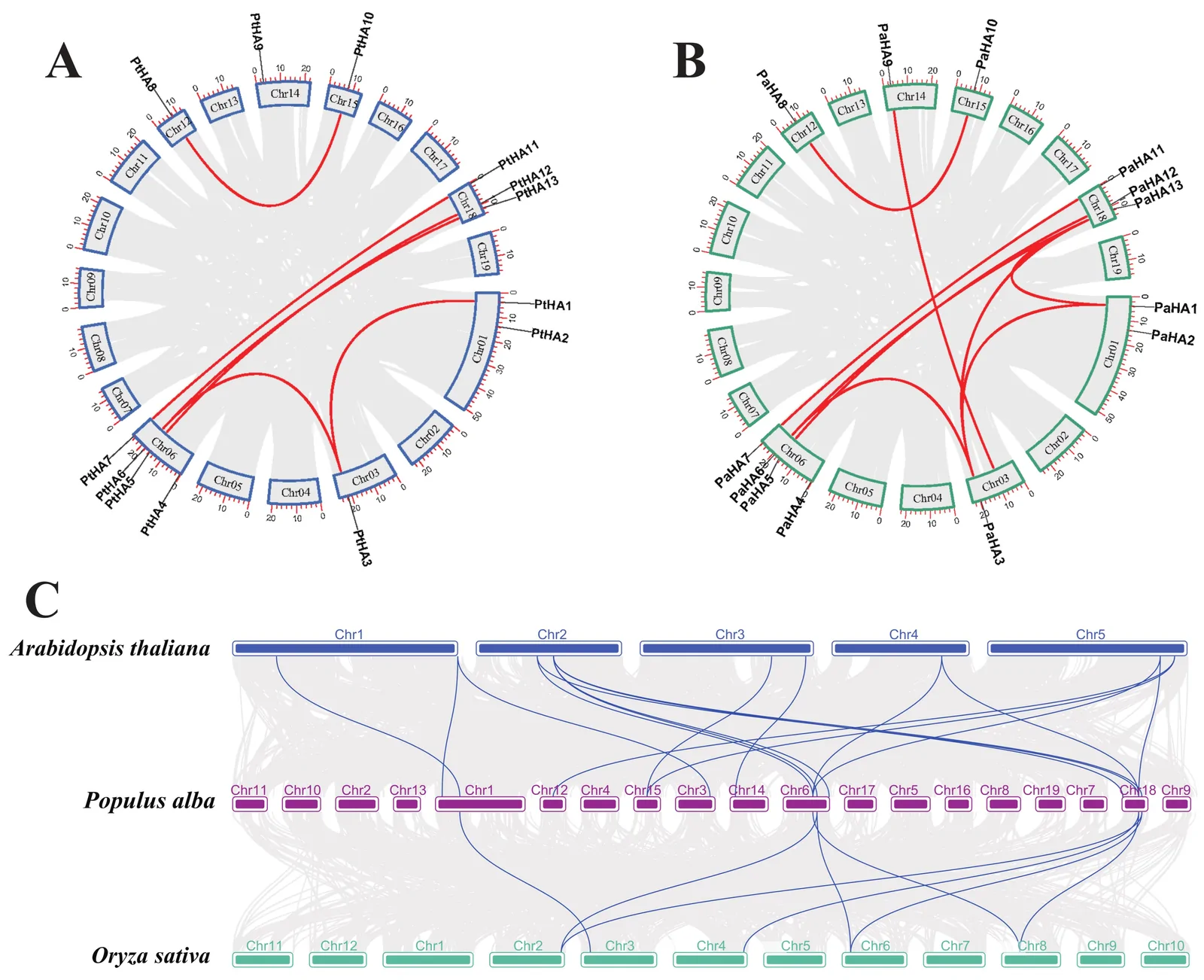
The Circos plots show the collinearity analysis of *HA* genes within the two haplotype genomes of 717 poplar (**A**,**B**), as well as inter-genomic collinearity analyses (**C**) between the *Pa* haplotype of 717 poplar and other plant species, namely *A. thaliana* and *O. sativa*. *Pt*, the *P. tremula* haplotype-resolved genome of 717 poplar; *Pa*, the *P. alba* haplotype-resolved genome of 717 poplar.

Synteny analyses were performed for each haplotype of the 717 poplar genome (Figure 3A,B). Six gene duplication events were identified among *PtHAs*, and nine duplication events were detected among *PaHAs* (Table S2 and Figure 3A,B). Shared duplication events between the two haplotypes included *HA8*/*HA10*, *HA7*/*HA11*, *HA6*/*HA3*, *HA3*/*HA1*, *HA12*/*HA5*, and *HA6*/*HA13* (Table S2 and Figure 3A,B). Eight syntenic gene pairs of *HA* genes were identified between the *Pa* haplotype of 717 poplar and rice, and 17 syntenic *HA* gene pairs were found between the *Pa* haplotype of 717 poplar and *A. thaliana* (Figure 3C). The Ka/Ks ratios of duplicated gene pairs in 717 poplar ranged from 0.049 to 0.193 (Table S2), suggesting that the *HA* gene family of 717 poplar has mainly undergone purifying selection during evolutionary divergence.

### 3.4. Cis-Acting Element Analysis of HAs in 717 Poplar

**Figure 4:**
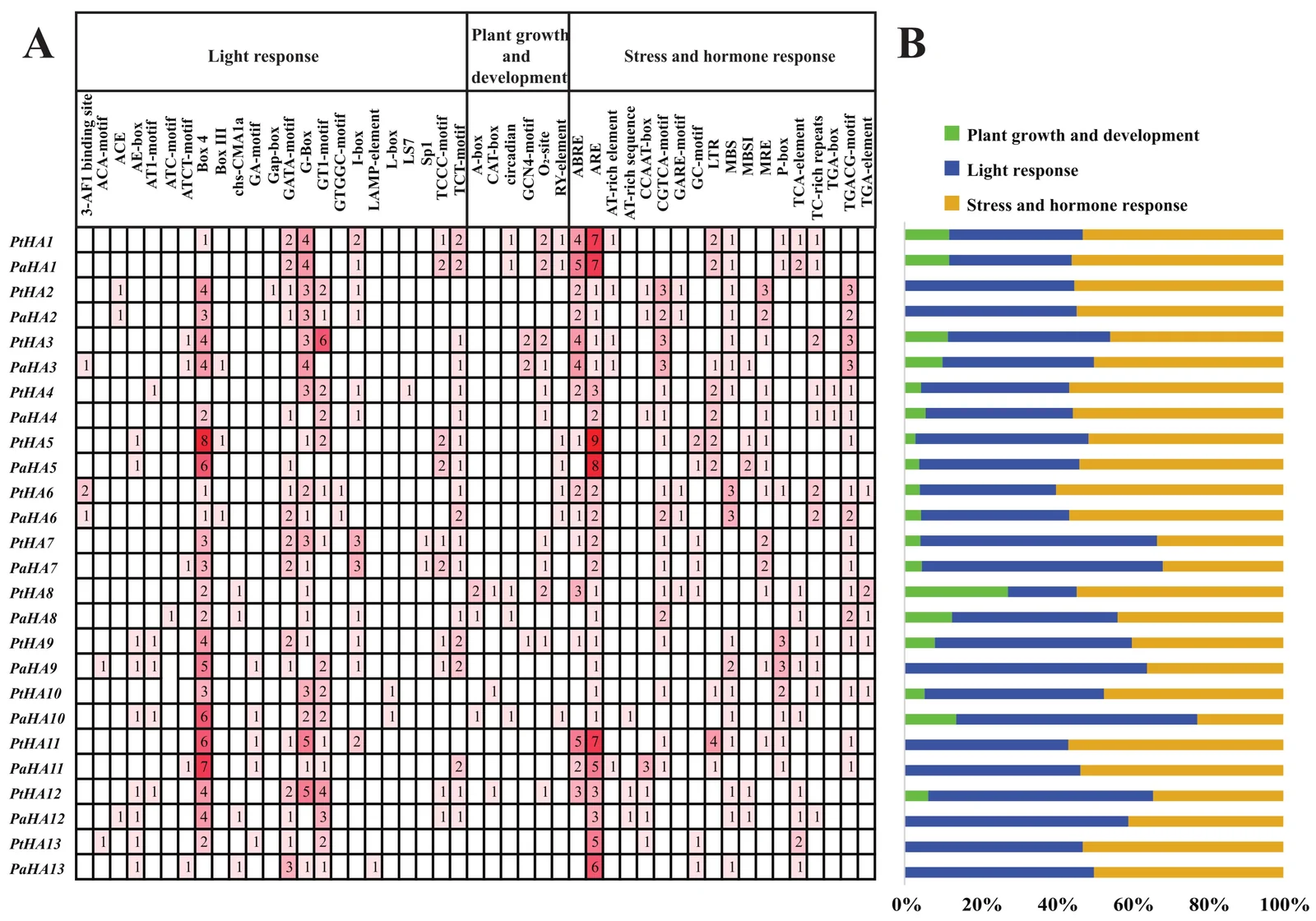
The number (**A**) and proportion (**B**) of *cis*-acting elements detected within the 2000 bp upstream regions of each identified *HA* gene in 717 poplar. The numbers inside cells and the depth of red color represent the counts of specific *cis*-acting elements for each *HA* gene. The proportions of different categories of *cis*-acting elements are presented as stacked bar charts. The gene prefixes *Pt* and *Pa* correspond to genes derived from the *P. tremula* and *P. alba* haplotypes of 717 poplar, respectively.

To investigate potential transcriptional regulatory patterns and functions of *HA* genes in 717 poplar, promoter sequences were extracted and subjected to *cis*-acting element annotation (Figure 4). Numerous *cis*-acting elements associated with plant growth and development, stress responses, hormone signaling, and light-responsive processes were identified within the promoters of *HA* genes in 717 poplar (Figure 4A). A variety of light-responsive elements, including AE-box and ATC-motif, were detected (Figure 4A). The light-responsive elements accounted for 18–64% of total *cis*-elements, whereas stress- and hormone-responsive elements occupied 23–60% (Figure 4B). Light-responsive elements such as BOX4 and GT1-motif were present in the promoters of most 717 poplar *HA* genes (Figure 4A), implying that *HA* genes may be regulated by light signaling. Within the stress- and hormone-responsive categories, stress-related elements including ABA-RESPONSIVE ELEMENT (ABRE), ANAEROBIC-RESPONSIVE ELEMENT (ARE) and MYB-BINDING SITE (MBS) were observed in most *HA* gene promoters (Figure 4A). ABRE and MBS are mainly involved in adaptation to abiotic stresses, and ARE mediates plant responses to hypoxia stress, suggesting that *HA* genes may contribute to plant tolerance to abiotic stresses. Multiple hormone-responsive elements, such as CGTCA-motif, TGACG-motif, and TCA-element, were also identified (Figure 4A), indicating that *HA* genes in 717 poplar can respond to diverse plant-hormone signals. Compared with the other two categories, fewer *cis*-acting elements related to plant growth and development were detected in promoters of 717 poplar *HA* genes, among which the RY-element (seed-specific regulation) and the circadian element were identified (Figure 4).

### 3.5. Expression Patterns and Stress-Responsive Analysis of the HA Gene Family in Poplar

**Figure 5:**
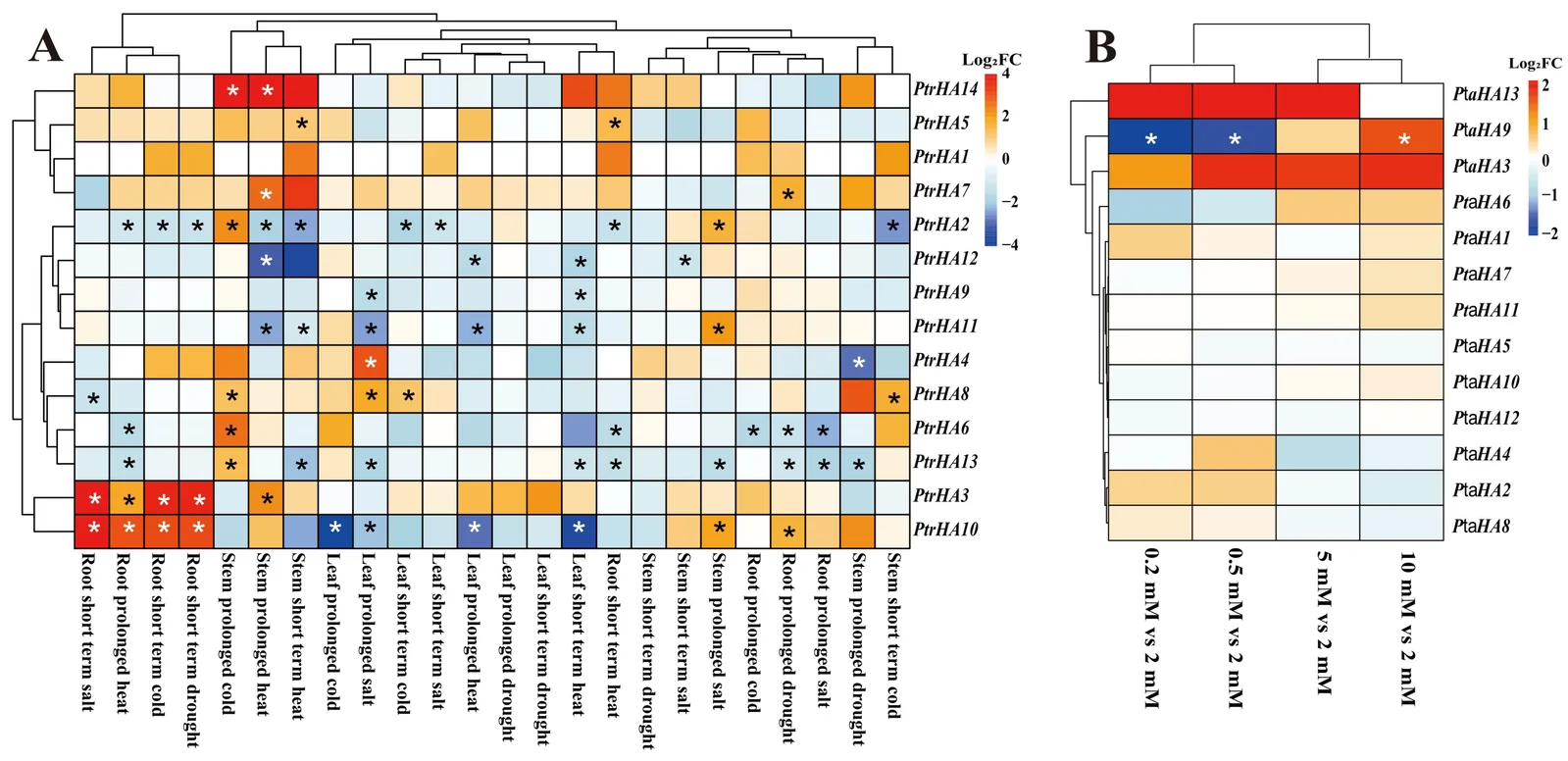
The heatmaps show the expression patterns of *PtrHAs* in different tissues and under stress treatments of *P. trichocarpa* (**A**), and the expression heatmap of *PtaHAs* under different nitrogen levels (**B**). The color gradient from blue to red in the heatmap indicates the log_2_FC values under given conditions relative to the control. Asterisks denote significant DEGs under the corresponding conditions, defined as |log_2_FC| > 1 and FDR < 0.05. *Ptr* for *P. trichocarpa*, *Pta* for *P. tremula* × *P. alba*.

Members of the *PtrHA* gene family exhibited highly tissue- and stress-specific transcriptional regulation in response to abiotic stresses in *P. trichocarpa* (Figure 5A). Among significantly up-regulated genes, *PtrHA14*, *PtrHA2*, *PtrHA6*, and *PtrHA13* were markedly activated in stems under long-term cold stress (Figure 5A). *PtrHA5* and *PtrHA7* exhibited distinct stress responses. *PtrHA5* was activated under short-term heat stress in roots and stems, while *PtrHA7* responded to both long-term drought stress in roots and long-term heat stress in stems (Figure 5A). *PtrHA4* was significantly up-regulated only in leaves under long-term salt stress (Figure 5A). *PtrHA8* was strongly induced under short-term cold stress in stems and leaves, long-term cold stress in stems, and long-term salt stress in leaves (Figure 5A). By contrast, *PtrHA3* and *PtrHA10* displayed broad-spectrum stress responses and were markedly up-regulated under long-term heat stress in roots as well as short-term treatments of three distinct stresses in roots (Figure 5A). Among significantly down-regulated genes, *PtrHA2* was repressed by heat, cold, salt, and drought stresses across multiple treatment durations in roots, stems, and leaves (Figure 5A). *PtrHA11* and *PtrHA12* were prominently down-regulated under various heat-stress conditions in stems and leaves (Figure 5A). *PtrHA4* was suppressed only under long-term drought stress in stems (Figure 5A).

According to the expression heatmap of *PtaHAs* under different nitrogen levels, only *PtaHA9* met the differential expression threshold among all *PtaHA* family members, whereas the remaining *PtaHA* genes did not exhibit expression changes that passed the statistical cutoff across nitrogen-treatment comparisons (Figure 5B). Relative to the 2 mM nitrogen control, *PtaHA9* was significantly down-regulated under 0.2 and 0.5 mM nitrogen treatments and markedly up-regulated under 10 mM nitrogen treatment (Figure 5B).

Expression levels of *PtrHAs* in *P. trichocarpa* across different growing seasons were retrieved from the Phytozome database (Figure S1). Based on expression-based clustering analysis, *PtrHA* family genes were divided into two clusters (Figure S1). The first cluster comprised *PtrHA8*, *PtrHA9*, *PtrHA13*, *PtrHA12*, *PtrHA2*, and *PtrHA11*, and all genes in this cluster exhibited relatively high expression in nearly all tested samples (Figure S1), indicating that these genes might be broadly involved in fundamental biological processes of *P. trichocarpa*. The second cluster consisted of *PtrHA10*, *PtrHA5*, *PtrHA7*, *PtrHA14*, *PtrHA6*, *PtrHA4*, *PtrHA1*, and *PtrHA3*, in which genes generally showed low expression levels, and transcripts of *PtrHA1* and *PtrHA3* were barely detectable in all examined tissues (Figure S1). Notably, *PtrHA5*, *PtrHA7*, and *PtrHA14* displayed extremely high transcript abundance in early- and mid-stage male-flower samples (Figure S1). In addition, *PtrHA10* showed high transcriptional abundance in apical buds at different dormancy-transition stages (Figure S1).

### 3.6. Co-Expression Network Analysis of the HA Gene Family in Poplar

**Figure 6:**
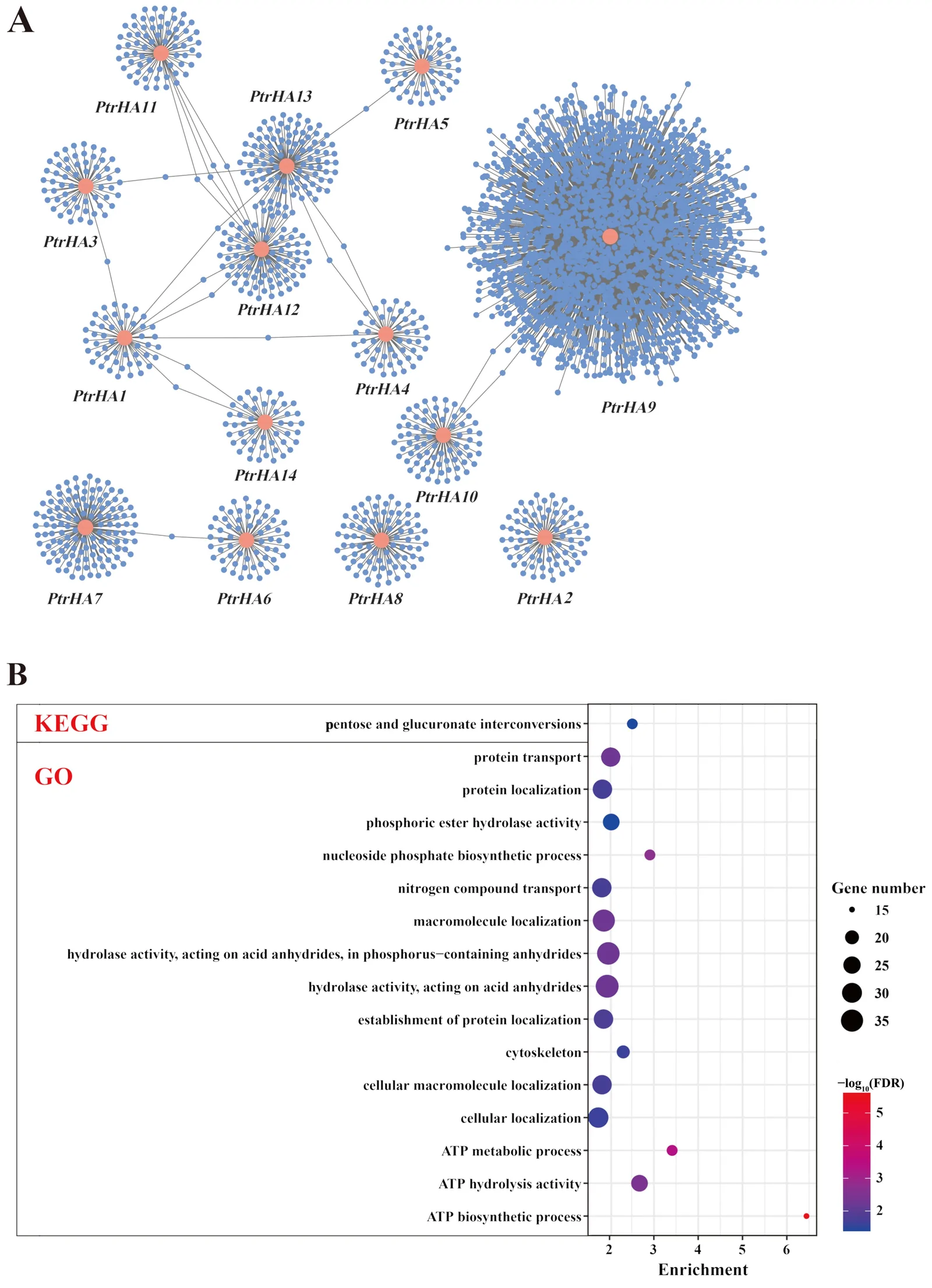
The co-expression network centered on *PtrHAs* (**A**), and GO and KEGG enrichment analyses of genes within this *PtrHA*-centered co-expression network (**B**). In the co-expression network, orange nodes represent *PtrHAs*, whereas blue nodes denote genes co-expressed with *PtrHAs* in *P. trichocarpa*.

To systematically decipher potential biological functions of the *PtrHA* gene family in poplar, a co-expression network centered on *PtrHA* genes in *P. trichocarpa* was constructed, which contained 14 *PtrHA* genes and 3022 co-expressed genes with 3037 co-expression relationships (Figure 6A). Among all core *PtrHA* genes, the co-expression subnetwork of *PtrHA9* was markedly larger than those of other *PtrHA* members, forming the largest co-expression gene module within the whole interaction network (Figure 6A). *PtrHA9* and *PtrHA10* shared partial co-expressed genes (Figure 6A), implying that they may cooperatively participate in identical biological regulatory pathways. Partial overlapping co-expressed genes were observed among *PtrHA1*, *PtrHA3*, *PtrHA4*, *PtrHA5*, *PtrHA11*, *PtrHA12*, *PtrHA13*, and *PtrHA14*; likewise, *PtrHA6* and *PtrHA7* shared one co-expressed gene (Figure 6A). In contrast, the co-expression subnetworks anchored by *PtrHA2* and *PtrHA8* existed independently, with no overlapping co-expressed genes with other *PtrHA*-mediated networks (Figure 6A). GO and KEGG functional enrichment analyses were performed on genes within the *PtrHA* co-expression network (Table S3 and Figure 6B). The results revealed that genes in this *PtrHA* co-expression network were enriched in carbohydrate metabolism, nucleotide metabolism, and energy metabolism processes, as well as in regulating biological processes, such as protein transport and localization, and various hydrolase activities (Table S3 and Figure 6B). These findings suggest possible functional associations with *PtrHAs*, which require further experimental validation.

## 4. Discussion

The *HA* gene family has been characterized in a wide range of plant species in recent years, including *Prunus persica* [67], rice [68], *B. napus* [69], *Malus pumila* [70], soybean [50], potato [71], *Gossypium hirsutum* [72], and *Pyrus betulaefolia* [73]. In this study, 26 *HA* genes were identified in 717 poplar, with 13 genes derived from each of the two haplotypes. All HA proteins were predicted to localize to the PM, which was consistent with the putative function of these proteins as transmembrane proton pumps. Protein structural analysis revealed that all HA proteins possess three conserved domains: e.g., haloacid dehalogenase-like hydrolase domain, E1–E2 ATPase domain, and cation transporter/ATPase N-terminus. These conserved domains are also present in HA proteins from woody plants such as apple and *P. betulaefolia* [70,73], suggesting potential conservation of core transmembrane proton-pump functions among dicot woody plants. Phylogenetic analysis divided the *HA* genes of 717 poplar into five subgroups—IA, IB, III, IIA, and IIB—representing the typical evolutionary classification of plant *HA* gene families. Gene duplication analysis revealed that duplicated gene pairs shared by the two haplotypes show obvious intra-subgroup clustering. Specifically, for 717 poplar, *HA8*/*HA10* belonged to subgroup IA; *HA7*/*HA11* and *HA12*/*HA5* belonged to subgroup IIB; *HA6*/*HA3*, *HA3*/*HA1*, and *HA6*/*HA13* belonged to subgroup IIA. Proteins encoded by these duplicated genes exhibit highly similar physicochemical properties, including amino acid sequence length, molecular weight, and isoelectric point. In line with this, similar results were also reported for *HA* gene families in woody plants such as cotton and apple [70,72]. Furthermore, a total of 17 collinear *HA* gene pairs were detected between the *Pa* haplotype of 717 poplar and the dicot *A. thaliana*, compared with only eight collinear pairs between the *Pa* haplotype of 717 poplar and rice. Consistent with the present results, previous studies on cotton, potato, and apple have illustrated that segmental duplication serves as the major driver for *HA* gene family expansion, and interspecies collinearity was closely correlated with phylogenetic distance [70,71,72].

*Cis*-acting elements in gene promoters are critical for modulating the spatiotemporal expression of genes and responses to external signals [74]. The promoters of *HA* genes harbored light-responsive elements (e.g., AE-box, GT1-motif), stress-responsive elements (e.g., ABRE, MBS), and hormone-responsive elements (e.g., CGTCA-motif). Light-responsive elements account for 18–64%, whereas stress- and hormone-related elements occupy 23–60% of the total *cis*-elements. These results suggested that *HA* gene expression was coordinately regulated by multiple signals including light, stress, and phytohormones. Notably, ARE elements were detected in the promoters of all *HA* genes from 717 poplar. ARE elements are essential for triggering anaerobic respiration [75]. AREs are also widely distributed across the *A. thaliana* genome and participate in ABA- and ethylene-mediated responses to multiple abiotic stresses such as anaerobiosis, drought, and salinity [76].

Saline stress represents a major abiotic stress that impairs plant growth and constrains ecosystem productivity worldwide [29,77]. Consistent with these results, significant up-regulation of *CsHA10* and *de novo* induction of *CsHA1* transcripts, which were undetectable in controls, have been reported in salt-stressed cucumber, and this response was accompanied by increased abundance of 14-3-3 proteins indicative of post-translational modification [78]. Mechanistically, 14-3-3 proteins activate the plasma membrane H^+^-ATPase by binding to its phosphorylated C-terminus (e.g., Thr^948^), thereby releasing auto-inhibition and enhancing proton-pump activities [79]. Such proton-pump activation provides the proton-motive force for ion transport, which, together with the high expression of the Na^+^/H^+^ antiporter SOS1, contributes to ion homeostasis under salt stress [80]. Further evidence from *P. euphratica*, a woody model species, demonstrates that WRKY transcription factors can bind to W-box elements in the *PeHA1* promoter, which up-regulates *PeHA1* transcription under salt stress and consequently elevates HA activities to improve plant salt tolerance [21]. In line with this, several *PtrHA* genes, such as *PtrHA3*, *PtrHA4*, *PtrHA8*, and *PtrHA10*, were significantly induced by salt stress in roots or leaves in *P. trichocarpa*. Collectively, *HA* genes play an indispensable and central role in mediating plant salt-stress responses.

Nitrogen is an essential macronutrient for poplar growth, and variations in nitrogen availability markedly alter plant metabolism and stress tolerance [40]. In this study, transcript abundance of *PtaHA9* varied markedly under different nitrogen supplies, showing significant down-regulation at 0.2 and 0.5 mM low nitrogen and strong induction at 10 mM high nitrogen relative to the 2 mM control. Notably, as CaCl_2_ was applied to balance calcium concentrations across nitrogen treatments, a chloride gradient inversely correlated with nitrate supply was inevitably produced; therefore, the potential effects of chloride supplementation cannot be fully excluded. These findings agree with a previous report on *P. popularis*, in which net root influxes of NH_4_^+^ and NO_3_^−^ are coupled with PM H^+^-ATPase activities [25]. Pharmacological assays have revealed that application of sodium vanadate, an HA inhibitor, reduces root nitrogen accumulation and whole-plant nitrogen accumulation in tea plants, indicating that HA activities are indispensable for nitrate uptake [81]. Similarly, rice elevates the expression of PM *HA* genes including *OSA1/3/7/8/9* to enhance H^+^ pumping for adaptation to low-pH environments, and the proton-motive force established by HA-driven H^+^ efflux serves as the driving power for ammonium transmembrane transport [82,83]. Co-expression network analysis revealed that 14 *PtrHA* genes formed a complex co-expression network with 3022 genes, among which *PtrHA9* possessed the largest co-expression module and shared partial co-expressed genes with *PtrHA10*, implying close co-expression relationships between these two genes. GO enrichment analysis demonstrated that co-expressed genes were predominantly enriched in pathways related to energy metabolism, carbohydrate metabolism, protein transport, and enzyme-activity regulation. HA catalyzes ATP hydrolysis to establish the transmembrane proton electrochemical gradient, which serves as the primary energy source across the plant PM and participates in diverse cellular substance-transport processes [84].

## 5. Conclusions

In the present study, 13 *HA* genes were identified from each haplotype of the 717 poplar, and these genes were distributed across seven chromosomes. All HA proteins in 717 poplar harbored three conserved domains. Six and nine segmental duplication events were detected for *PtHAs* and *PaHAs*, respectively. The promoters of *PtHA* and *PaHA* genes contained abundant *cis*-acting elements associated with light, phytohormones, and abiotic stresses. Transcriptomic analyses revealed that the expression of *PtrHA* genes was modulated by various stresses. Furthermore, *PtaHA9* from 717 poplar was down-regulated under low-nitrogen conditions, while the opposite pattern was observed under high-nitrogen conditions, implying a certain association with nitrogen availability. These expression patterns highlight *PtaHA9* as a promising candidate for further characterization of its nitrogen response. Co-expression network analysis showed correlations between *PtrHAs* and co-expressed genes involved in carbohydrate and energy metabolism as well as protein transport, pointing to putative functional associations of *PtrHAs* in poplar physiological metabolism and stress regulation. These findings provide a fundamental resource for further functional characterization of poplar *HA* family genes and the screening of stress-tolerance candidate genes.

## Data Availability

The raw RNA-seq data supporting the findings of this study are available in the Genome Sequence Archive (GSA) of the National Genomics Data Center (NGDC), with BioProject accession PRJCA074346.

## Supplementary Materials

The following supporting information can be downloaded at https://www.mdpi.com/article/10.3390/life16101691/s1, Figure S1: *PtrHAs* expression in different tissues under various conditions; Table S1: Basic information of *HA* genes in 717 poplar; Table S2: Ka/Ks analysis of gene pairs; Table S3: The GO and KEGG analyses.
