# Supplementary material for: Genome-Wide Identification of the Plasma Membrane H+-ATPase in Poplar and Their Responses to Abiotic Stresses and Nitrogen Availability
Source: Life (Basel). 2026 Oct 9;16(10):1691. doi: 10.3390/life16101691 (PMC13706358; doi:10.3390/life16101691)
Supplement: Supplementary file 1 [file life-16-01691-s001.zip › Figure S1.pdf]

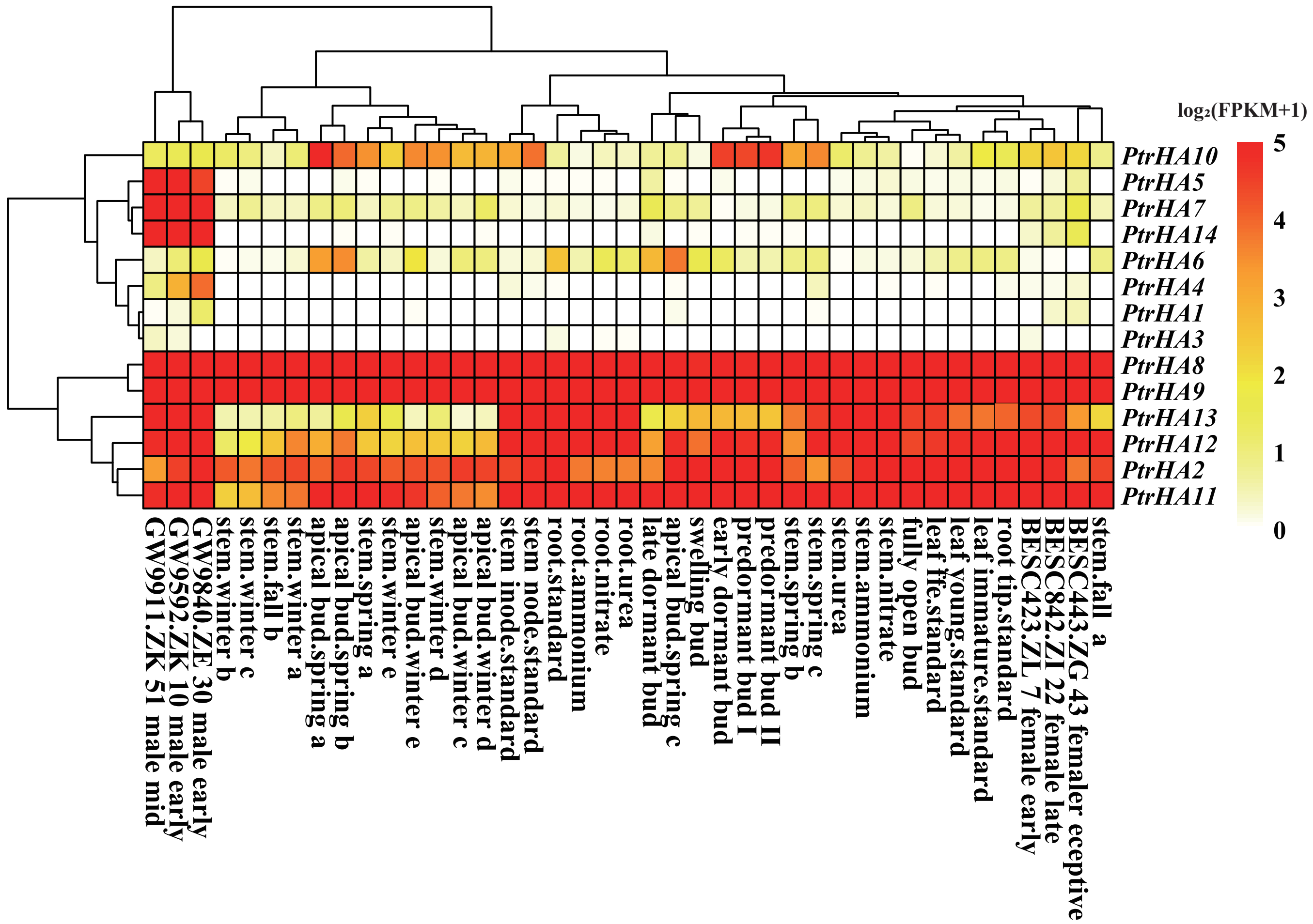

Figure S1. PtrHAs expression in different tissues under various conditions.
